# The novel cytotoxic polybisphosphonate osteodex decreases bone resorption by enhancing cell death of mature osteoclasts without affecting osteoclastogenesis of RANKL-stimulated mouse bone marrow macrophages

**DOI:** 10.1007/s10637-024-01427-1

**Published:** 2024-03-01

**Authors:** Petra Henning, Anna Westerlund, Sofia Movérare-Skrtic, Catharina Lindholm, Marcela Márquez-Méndez, Sten Nilsson, Anders R. Holmberg, Ulf H. Lerner

**Affiliations:** 1 Centre for Bone and Arthritis Research at Institute of Medicine, Sahlgrenska Osteoporosis Centre, Sahlgrenska Academy at the University of Gothenburg, Vita Stråket 11, Gothenburg 41345, Sweden; 2 Department of Oncology and Pathology, Karolinska Institute, Stockholm SE-171 76, Sweden; 3 Molecular Periodontology, Faculty of Medicine, Umeå University, SE-901 87 Umeå, Sweden

**Keywords:** Bisphosphonates, Osteodex, Osteoclasts, RANKL

## Abstract

It has previously been demonstrated that the polybisphosphonate osteodex (ODX) inhibits bone resorption in organ-cultured mouse calvarial bone. In this study, we further investigate the effects by ODX on osteoclast differentiation, formation, and function in several different bone organ and cell cultures. Zoledronic acid (ZOL) was used for comparison. In retinoid-stimulated mouse calvarial organ cultures, ODX and ZOL significantly reduced the numbers of periosteal osteoclasts without affecting *Tnfsf11* or *Tnfrsf11b* mRNA expression. ODX and ZOL also drastically reduced the numbers of osteoclasts in cell cultures isolated from the calvarial bone and in vitamin D3–stimulated mouse crude bone marrow cell cultures. These data suggest that ODX can inhibit osteoclast formation by inhibiting the differentiation of osteoclast progenitor cells or by directly targeting mature osteoclasts. We therefore assessed if osteoclast formation in purified bone marrow macrophage cultures stimulated by RANKL was inhibited by ODX and ZOL and found that the initial formation of mature osteoclasts was not affected, but that the bisphosphonates enhanced cell death of mature osteoclasts. In agreement with these findings, ODX and ZOL did not affect the mRNA expression of the osteoclastic genes *Acp5* and *Ctsk* and the osteoclastogenic transcription factor *Nfatc1*. When bone marrow macrophages were incubated on bone slices, ODX and ZOL inhibited RANKL-stimulated bone resorption. In conclusion, ODX does not inhibit osteoclast formation but inhibits osteoclastic bone resorption by decreasing osteoclast numbers through enhanced cell death of mature osteoclasts.

## Introduction

Bisphosphonates (BPs) are anti-resorptive pharmaceuticals that have been used for several decades in the treatment of diseases with excessive formation of osteoclasts like osteoporosis, skeletal metastasis of malignant tumors, and malignant osteolysis with hypercalcemia [1]. The efficacy of BPs as inhibitors of bone resorption and skeletal fractures is demonstrated by the findings that a yearly intravenous administration of zoledronic acid to postmenopausal women reduced all clinical fractures by 35% during a 2-year follow-up [2] and new vertebral fractures and hip fractures by 70 and 41%, respectively, during a 3-year follow-up [3]. According to the “seed-and-soil” hypothesis, suggested by Paget more than 100 years ago [4], osteoclasts provide tumor growth substances from the bone matrix during bone resorption in osteolytic lesions in patients with breast and lung cancer, as well as in sclerotic lesions in patients with prostatic cancer [5–7]. For this reason, BPs are used with the aim not only to protect the skeleton from excessive bone resorption and skeletal-related events (SRE) but also to reduce tumor growth [8]. Zoledronic acid (ZOL) has been shown to significantly reduce the time to first SRE and the overall risk of SRE in breast cancer patients with skeletal metastases [9] and in patients with skeletal metastasis of lung cancer and other solid tumors except breast and prostatic cancers [10, 11].

The general chemical structure of BPs is two phosphonate groups linked to a central carbon (P–C-P) and with two sidechains, R^1^ and R^2^, linked to the central carbon [1, 12]. The P–C-P group renders the compounds resistant to degradation by phosphatases. The R^1^ side group is usually a hydroxyl group facilitating binding to hydroxyapatite crystals in bone, and R^2^ may have a range of chemical structures. There are two classes of BPs, nitrogen-containing and those without nitrogen, where those having nitrogen in the R^2^ side chain are second- and third-generation BPs. BPs containing nitrogen are several orders of magnitude more potent as anti-resorptive agents than the first-generation BPs [13]. Third-generation BPs are the most potent compounds with a tertiary nitrogen incorporated within a ring structure, e.g., imidazole in ZOL. BPs bound to bone become internalized in osteoclasts [14] when these cells resorb bone, which causes accumulation of BPs at concentrations high enough to inhibit osteoclast activity through apoptotic cell death [15, 16].

The current prevailing hypothesis regarding the primary mode of action, i.e., how nitrogen-containing BPs inhibit osteoclastic bone resorption, is by inhibition of farnesyl diphosphate synthase (FPPS), an enzyme in the mevalonate pathway [12]. The ultimate consequence of FPPS inhibition is loss of geranylgeranylated GTPases leading to disruption of osteoclast cytoskeleton, osteoclast apoptotic cell death, and loss of bone resorption activity [17], although the intracellular mechanism leading to the pro-apoptotic pathway is unknown.

Osteodex (ODX) is a polymer conjugate constituting a carbohydrate backbone with alendronate and guanidine moieties covalently coupled to the backbone. ODX is bifunctional, having anti-resorptive properties and pronounced anti-tumor efficacy [18]. We have previously reported that ODX inhibits bone resorption in organ-cultured mouse calvarial bones [18]. The aim of the present study was to explore the cellular mechanism by which ODX inhibits bone resorption. To achieve this, we utilized various bone organ and bone cell culture systems that allowed us to study osteoclast differentiation, formation, and function.

## Materials and methods

### Materials

Recombinant mouse macrophage colony-stimulating factor (M-CSF) and recombinant extracellular domain of mouse receptor activator of NF-κB ligand (RANKL) (Arg72-Asp316) fused to a six histidine residue tag (cat. no. 462-TR) were purchased from R&D Systems; the kit for leukocyte acid phosphatase staining, SIGMA 104 Phosphatase Substrate, ATRA, and zoledronic acid were from Sigma Chemical Co. (www.sigmaaldrich.com); α-modification of minimum essential medium (α-MEM), and fetal calf serum (FCS) were from Thermo Fisher Scientific; Thermo Sequenase™ II DYEnamic ET™ terminator cycle sequencing kit were from Amersham (www.amersham.com); oligonucleotide primers were from Invitrogen (www.invitrogen.com) or Applied Biosystems (www.appliedbiosystems.com); HotStarTaq polymerase kit and QIAquick PCR Purification Kit were from QIAGEN Ltd. (www.qiagen.com); DNA free was obtained from Ambion, Inc. (www.ambion.com); 1st strand cDNA synthesis kit and the PCR Core Kit were from Roche (www.roche-applied-science.com); fluorescent-labeled probes (reporter fluorescent dye VIC at the 5′end and quencher fluorescent dye TAMRA at the 3′end), TaqMan Universal PCR Master Mix, and the kits for quantitative real-time PCR were from Applied Biosystems (www.appliedbiosystems.com); culture dishes, multiwell plates, and glass Chamber Slides were from Nunc Inc. (www.nuncbrand.com); suspension culture dishes were from Corning Inc. (www.scienceproducts.corning.com); and bone slices and CrossLaps® for Culture ELISA (CTX) were from Immunodiagnostics a/s (www.idsplc.com/no/home/).

1,25(OH)_2_-vitamin D3 (D3) was a kind gift from Hoffmann-La Roche, Basle, Switzerland. ODX was a kind gift from DexTech Medical, Uppsala, Sweden. The cathepsin K antiserum was a kind gift from Professor Göran Andersson at Karolinska Institute, Stockholm, Sweden.

### Animals

We utilized CsA mice from our own inbred colony at Umeå University to conduct bone organ cultures, periosteal cell cultures, and crude bone marrow cell cultures. These mice have been extensively used in numerous studies for over 30 years, and the results obtained have always been comparable to those seen in other mouse strains, including C57BL/6 mice. C57BL/6 mice from Harlan Laboratories, Inc., and Taconic Bioscience were used for the bone marrow macrophage cultures. We ensured that animal care and experiments were conducted in accordance with internationally accepted standards of humane animal care. Additionally, we used animals only as deemed appropriate by the Animal Care and Use Committees of Umeå University, Umeå, and the University of Gothenburg, Gothenburg.

### Mouse calvarial bone cultures

Parietal bones from 5- to 7-day-old mice were microdissected and cut into calvarial halves. The bones were preincubated for 18–24 h in α-MEM containing 0.1% albumin and 1 µmol/l indomethacin [19, 20]. Following preincubation, the bones were extensively washed and subsequently cultured for 96 h in multiwell culture dishes containing 1.0 ml of an indomethacin-free medium with or without test substances. The bones were incubated in the presence of 5% CO_2_ in humidified air at 37 °C. At the end of the cultures, bones were used for immunohistochemistry or gene expression analysis.

### Mouse calvarial periosteal cell cultures

Cells were isolated from 2- to 5-day-old mice using time-sequential collagenase digestion, and cells from all digestions (1–10) were pooled [21]. These isolations contain not only osteoblastic cells but also osteoclast progenitor cells [21, 22]. The periosteal cells were seeded in 2 cm^2^ multiwell dishes at a density of 10^3^ cells/cm^2^ and incubated in α-MEM/10% FCS in the absence or presence of RANKL with or without either ODX or ZOL for 12 days. At the end of the cultures, the cells were stained for tartrate-resistant acid phosphatase (TRAP), and cells with more than three nuclei, expressing TRAP, were considered osteoclasts and numbers counted (TRAP^+^MuOCL).

### Mouse bone marrow cell cultures

Bone marrow cells (BMC) were flushed from femurs and tibiae from 5- to 7-week-old male mice. BMC were seeded in 48 multiwells (10^6^ cells/cm^2^), incubated overnight in α-MEM/10% FCS, and subsequently cultured in the same medium in the absence or presence of 1,25(OH)_2_-vitamin D3, with or without ODX or ZOL for 9 days. After this time period, the cells were fixed with acetone in citrate buffer/3% formaldehyde and stained for TRAP. TRAP-positive cells with three or more nuclei were considered osteoclasts and the number of multinucleated osteoclasts was counted (TRAP^+^MuOCL).

### Mouse bone marrow macrophage cultures

Bone marrow cells were flushed from femur and tibiae and seeded in α-MEM/10% FCS containing 30 ng/ml mouse M-CSF on plastic suspension culture dishes to which stromal cells and lymphocytes do not adhere [21, 23]. After 2 days, the adhering cells (bone marrow macrophages (BMM)) were detached, and then, 5000 cells in 5 µl α-MEM/10% FBS were spot seeded at the center of 96-well plates and left to adhere for 10 min. Subsequently, 200 µl medium was added containing either 30 ng/ml of M-CSF (controls) or 30 ng/ml M-CSF + 4 ng/ml of RANKL, without and with ODX or ZOL. After 3–4 days, the cells were fixed and stained for TRAP. TRAP-positive cells with three or more nuclei were considered osteoclasts, and multinucleated osteoclasts (TRAP^+^MuOCL) were counted. For the actin ring staining, osteoclasts were fixed after 4 days with 4% phosphate-buffered formaldehyde for 20 min, washed 3 times in PBS, and permeabilized using 0.1% Triton X-100 for 10 min. Then, the cells were incubated with 2% BSA/PBS and stained with FITC-labeled phalloidin diluted 1:40 in 2% BSA/PBS for 30 min. In some experiments, 20,000 cells in 20 µl α-MEM/10% FBS were spot-seeded in 48-well plates, cultured as above, and used for gene expression analysis.

Mouse BMM were also seeded on slices of devitalized bovine bone (2 × 10^4^ cells/bone slice) in 96-well plates in α-MEM/10% FCS and cultured for up to 14 days, with change of medium every third day. Subsequently, cells were removed, and bones were stained with 0.5% toluidine blue to visualize resorption pits. The release of CTX into the culture medium during resorption was analyzed by CrossLaps ELISA.

### Immunohistochemistry

Calvarial bones were fixed in 4% phosphate-buffered paraformaldehyde; decalcified in 10% EDTA in Tris buffer, pH 6.95; and embedded in paraffin. Sections were cut, deparaffinized, fixed in cold acetone, and subsequently treated with 3% H_2_O_2_ in PBS and Avidin/Biotin blocking kit. After blocking with protein block, sections were incubated with unlabeled polyclonal rabbit anti-mouse cathepsin K [24] diluted 1:700 or normal rabbit serum as a negative control. After blocking with normal goat serum, biotin-labeled goat anti-rabbit serum was used as a secondary antibody and was followed by incubation with a VECTASTAIN ABC kit and DAB substrate kit. All sections were counterstained with Mayer’s hematoxylin and evaluated using a Leica Q500MC microscope (Leica, Cambridge, UK) by an observer (CL) blinded to the identity of the sections. The numbers of cathepsin K–positive multinucleated cells per section were determined; two sections per bone were analyzed.

### RNA extraction and gene expression

RNA was isolated from mouse calvarial bone cultures using the RNAqueous-4 PCR kit. Single-stranded cDNA was synthesized from 0.1 to 0.5 µg of total RNA using a High Capacity cDNA Reverse Transcription Kit. Quantitative real-time PCR analysis of *Tnfsf11* and *Tnfrsf11b* was performed using the KAPA™ Probe Fast qPCR Kit with primers and probe as described in detail previously [25].

RNA from bone marrow macrophage cultures was isolated using the RNeasy Micro Kit. Single-stranded cDNA was synthesized using a High Capacity cDNA Reverse Transcription Kit, and gene expression was analyzed using custom TaqMan Assays. The following premade primer–probe mix from Applied Biosystems assays was used: *Acp5* (Mm00475698_m1), *Ctsk* (Mm00484036_m1), *Nfatc1* (Mm00479445_m1), *Fas* (Mm01204974_m1), *Bax* (Mm00432051_m1), *Bcl2* (Mm00477631_m1), *Bcl2l1* (Mm00437783_m1). The housekeeping gene 18S was used as endogenous control, and the data were displayed as percent of control.

### Statistics

Statistical differences were analyzed using one-way ANOVA, followed by Dunnett’s multiple comparisons test versus ATRA, D3, RANKL, or M-CSF/RANKL treated cells as indicated.

## Results

### Osteodex decreases osteoclast numbers in mouse calvarial bones

**Fig. 1:**
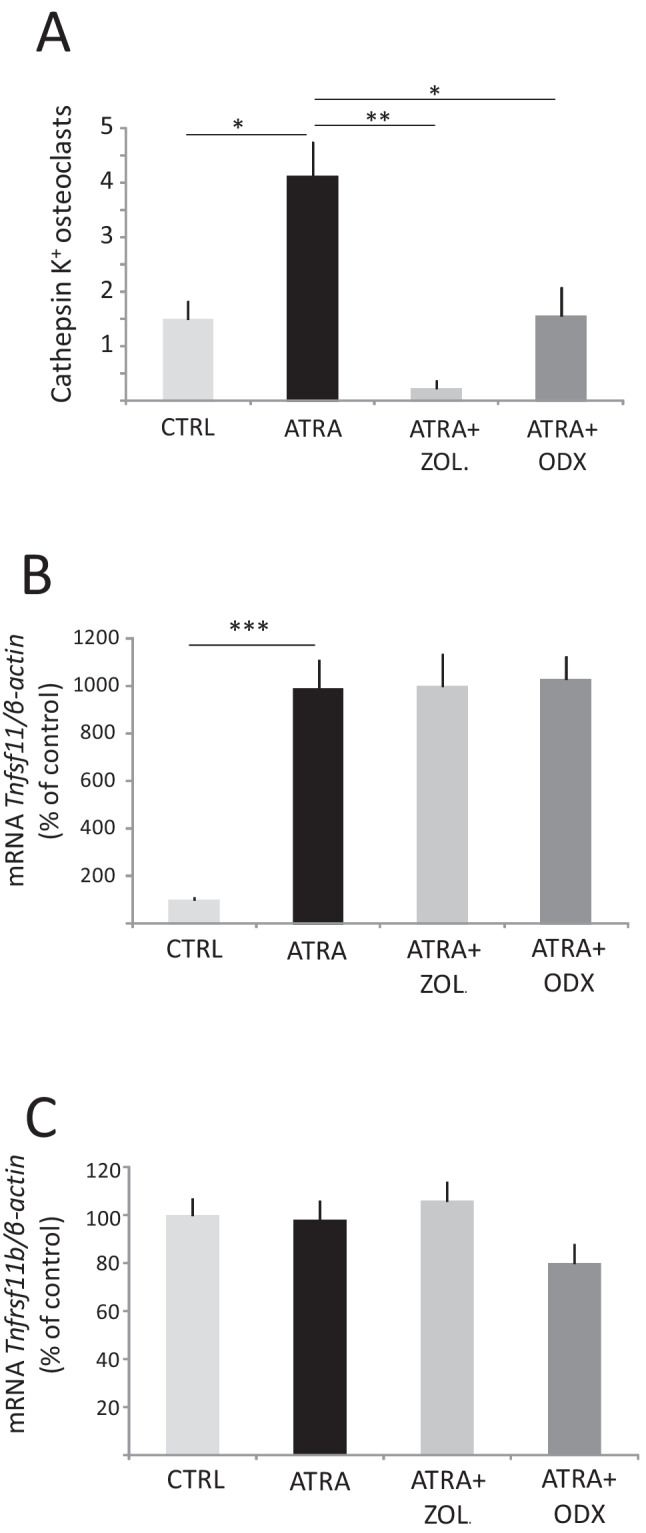
Osteodex (ODX) and zoledronic acid (ZOL) decrease osteoclast numbers induced by all-*trans*-retinoic acid (ATRA) in cultured neonatal mouse calvarial bones (**A**) without affecting the mRNA expression of the osteoclastogenic cytokine *Tnfsf11* or its decoy inhibitor *Tnfrsf11b* (**B**, **C**). Calvarial explants were incubated in the presence of ATRA (10^−7^ M) with or without ODX or ZOL, both at 2 × 10^−7^ M for 96 h, and numbers of osteoclasts per section were counted after immunostaining for cathepsin K (**A**). Gene expression of *Tnfsf11* and *Tnfrsf11b* was analyzed after 48 h (**B**, **C**). Data are means of four observations, and SEM is given as vertical bars. Asterisks denote statistical significance; *P < 0.05, **P < 0.01, and ***P < 0.001, one-way ANOVA, followed by Dunnett’s multiple comparisons test versus ATRA

We have previously shown that ODX can inhibit bone resorption, as assessed by mineral release, in organ-cultured mouse calvarial bones stimulated by all-*trans*-retinoic acid (ATRA) [18], a well-known stimulator of bone resorption in vitro and in vivo [26]. To determine whether inhibition of bone resorption by ODX was a result of decreased numbers of osteoclasts, we stimulated bone resorption in the neonatal mouse calvarial bones in ex vivo organ cultures by using ATRA (10^−7^ M) [27] with and without either ODX (2 × 10^−7^ M) or ZOL (2 × 10^−7^ M). ZOL is the most potent, clinically used nitrogen-containing BP inhibiting bone resorption by causing osteoclast apoptosis [28] and used in the present experiments as a positive control. ODX and ZOL significantly decreased the numbers of cathepsin K^+^ osteoclasts present in the periosteum of the calvarial bones after ATRA treatment (Fig. 1A).

In the calvarial bones, the inhibitors might act either at the level of RANKL-producing osteoblasts or by directly targeting the osteoclasts or their progenitors. We, therefore, assessed if ODX affected the ATRA-induced expression of RANKL and its inhibitor OPG. Neither ODX nor ZOL affected the robust enhancement of *Tnfsf11* mRNA expression (encoding RANKL) induced by ATRA (Fig. 1B). The mRNA expression of *Tnfrsf11b* (encoding OPG) was not affected by ATRA, in agreement with previous observations [27], and the expression was not affected by co-treatment with ODX or ZOL (Fig. 1C).

### Osteodex decreases the numbers of osteoclasts in mouse calvarial periosteal cell cultures

**Fig. 2:**
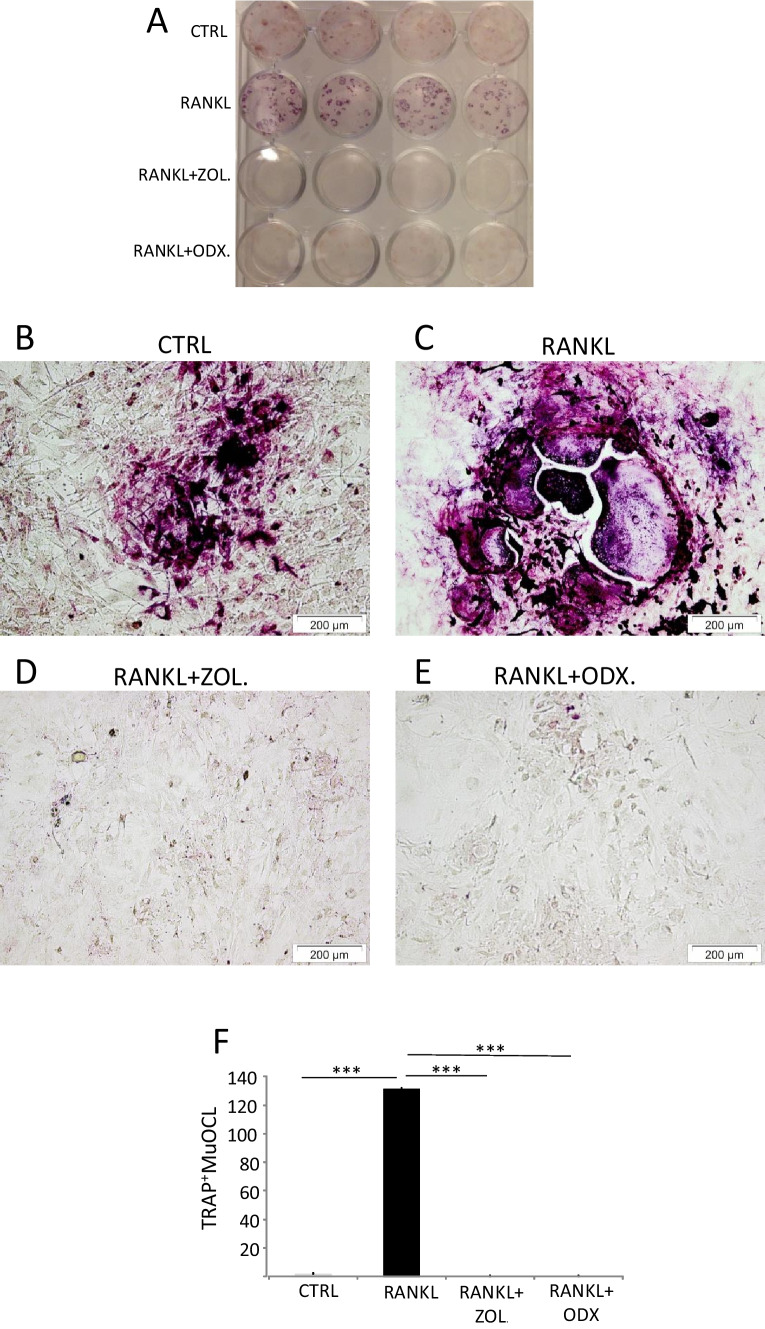
Osteodex (ODX) and zoledronic acid (ZOL) decrease osteoclast numbers induced by RANKL in mouse calvarial periosteal cell cultures. Cells were isolated from neonatal mouse calvaria and incubated in the presence of RANKL (10 ng/ml) with or without ODX or ZOL, both at 2 × 10^−7^ M for 12 days and then stained for TRAP. Overview photo of TRAP-stained cells in culture plate (**A**), representative microscope photos (**B**), and counting of TRAP^+^MuOCL (**C**). Data are means of four observations, and SEM is given as vertical bars. Asterisks denote statistical significance; ***P < 0.001, one-way ANOVA, followed by Dunnett’s multiple comparisons test versus RANKL-treated cells

The osteoclasts formed in the mouse organ cultured bones are derived from mononuclear osteoclast progenitors present in the periosteum. We have reported that such progenitors are present in collagenase-digested periosteal cell isolations from neonatal mouse calvarial bones and that stimulation of cells isolated from the periosteum results in mature osteoclast formation [21]. As shown in Fig. 2A, B, TRAP^+^ cells were formed in unstimulated control cultures, but very few were TRAP^+^MuOCL (Fig. 2F). Stimulation of the cell cultures with RANKL (10 ng/ml) for 12 days resulted in the formation of many TRAP^+^MuOCL (Fig. 2A, C), and this response was abolished by ODX and ZOL (Fig. 2A, D–F).

### Osteodex decreases the numbers of osteoclasts in mouse bone marrow cell cultures

**Fig. 3:**
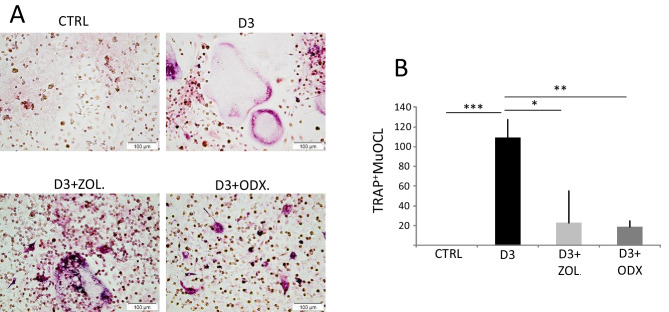
Osteodex (ODX) and zoledronic acid (ZOL) decrease osteoclast numbers induced by 1,25(OH)2-vitamin D3 (D3) in mouse bone marrow cell cultures. Mouse bone marrow cells were incubated in the absence or presence of D3 (10^−8^ M) with or without ODX or ZOL, both at 2 × 10^−7^ M for 9 days and then stained for TRAP. Representative microscope photos (**A**) and counting of TRAP^+^MuOCL (**B**). Data are means of four observations, and SEM is given as vertical bars. Asterisks denote statistical significance; *P < 0.05, **P < 0.01, and ***P < 0.001, one-way ANOVA, followed by Dunnett’s multiple comparisons test versus D3

Bone marrow cell cultures are widely used to assess osteoclast formation. To explore the potential of ODX in inhibiting the formation of osteoclasts from bone marrow osteoclast progenitor cells, we stimulated crude mouse bone marrow cells (BMC) cultures with 1,25(OH)_2_-vitamin D3 (D3). D3 primarily targets stromal cells present in the BMC cultures inducing their expression of RANKL, and subsequently, the differentiation of RANK^+^ osteoclast progenitors is stimulated [29]. Stimulation of BMC cultures with D3 (10^−8^ M) for 6 days resulted in the formation of many TRAP^+^MuOCL (Fig. 3A, B). Co-treatment with either ODX or ZOL substantially decreased the number of TRAP^+^MuOCL (Fig. 3A, B).

### Osteodex enhances osteoclast cell death without affecting osteoclast differentiation in bone marrow macrophage cultures

The observations in the calvarial bone and BMC cultures suggest that ODX decreases the numbers of osteoclasts either by inhibiting osteoclast progenitor cell differentiation or fusion at late stages or by acting directly on mature osteoclasts to enhance cell death. To further assess if ODX can directly target osteoclast progenitor cells, we used purified bone marrow macrophage (BMM) cultures, which were stimulated by M-CSF and RANKL to induce osteoclastogenesis. We then analyzed the effect by ODX and ZOL either by analyzing osteoclast differentiation in TRAP-stained cultures at different time points or by analyzing the expression of osteoclastic and osteoclastogenic genes.

**Fig. 4:**
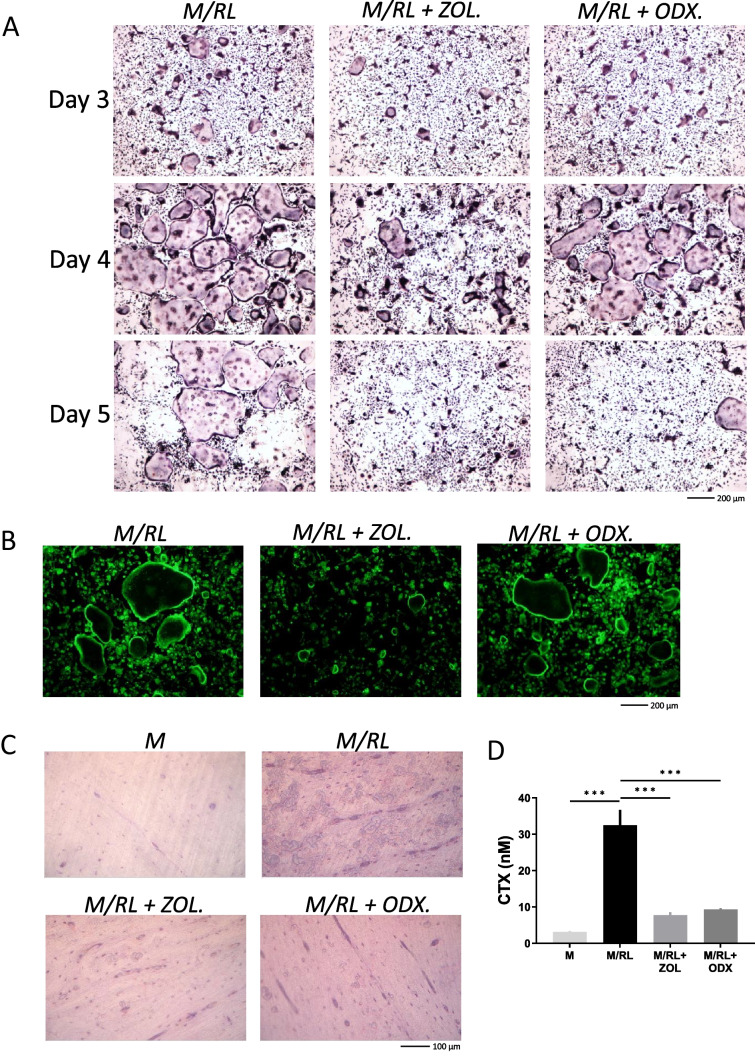
Osteodex (ODX) and zoledronic acid (ZOL) do not affect osteoclast formation in bone marrow macrophage (BMM) cultures but enhance mature osteoclast cell death and inhibit late stages of bone resorption. BMMs were purified from bone marrow cells and then incubated in the presence of M-CSF (M; 30 ng/ml) and RANKL (RL; 4 ng/ml) with or without ODX or ZOL, both at 2 × 10^−7^ M. At the stated time periods, cells were stained for TRAP (**A**) or with FITC-labeled phalloidin (**B**). In separate experiments, BMM were incubated on bone discs and resorption pits visualized by toluidine blue staining and reflective light microscopy after 14 days (**C**), and CTX released to culture medium during days 10–14 was analyzed (**D**). Data are means of four observations, and SEM is given as vertical bars. Asterisks denote statistical significance; ***P < 0.001, one-way ANOVA, followed by Dunnett’s multiple comparisons test versus M/RL

In BMM cultures stimulated by M-CSF (30 ng/ml) and RANKL (4 ng/ml) for 3 days, most of the mononucleated cells were TRAP^+^, and some of them had formed TRAP^+^MuOCL (Fig. 4A). After 4 days, very many of the BMM stimulated by M-CSF/RANKL had formed mature TRAP^+^MuOCL (Fig. 4A), and 1 day later, several of these cells had started to die as assessed by their morphology (Fig. 4A). Treatment of the M-CSF/RANKL-stimulated BMM with ODX (2 × 10^−7^ M) did not affect the appearance of mononucleated TRAP^+^ cells or mature TRAP^+^MuOCL at day 3 or 4 (Fig. 4A). At day 5, however, very few mature TRAP^+^MuOCL could be seen (Fig. 4A). Similar to ODX, treatment with ZOL (2 × 10^−7^ M) did not affect the appearance of TRAP^+^ mono- or multinucleated osteoclasts at day 3 (Fig. 4A). At day 4, the numbers of mature TRAP^+^MuOCL were fewer in ZOL-treated cultures than in M-CSF/RANKL-stimulated BMM with or without ODX (Fig. 4A). At day 5, no mature TRAP^+^MuOCL could be seen in the ZOL-treated cells similar to the observation in ODX-treated BMM. In agreement with these findings, ODX did not affect the presence of mature osteoclasts with phalloidin^+^ actin rings at day 4, whereas these cells were much fewer in ZOL-treated BMM cultures (Fig. 4B).

To investigate if ODX can inhibit bone resorption when BMM cells were cultured on bone slices, we incubated BMM on bovine bone slices for 14 days in the presence of M-CSF/RANKL with or without ODX or ZOL. The formation of osteoclasts in BMM cultures on bone slices was considerably delayed compared to BMM cultures on plastic dishes, and therefore, bone resorption was assessed by analyzing the release of the bone matrix fragment CTX from the bones to the media during days 10 to 14 and bone resorption pits visualized by toluidine blue staining on day 14. Stimulation of BMM with M-CSF/RANKL resulted in the formation of numerous resorption pits, a response that was substantially reduced by ODX or ZOL (Fig. 4C). The release of CTX was enhanced tenfold (approx.) from bone slices with BMM stimulated by M-CSF/RANKL compared to M-CSF-stimulated controls (Fig. 4D). This response was substantially decreased by ODX and ZOL.

**Fig. 5:**
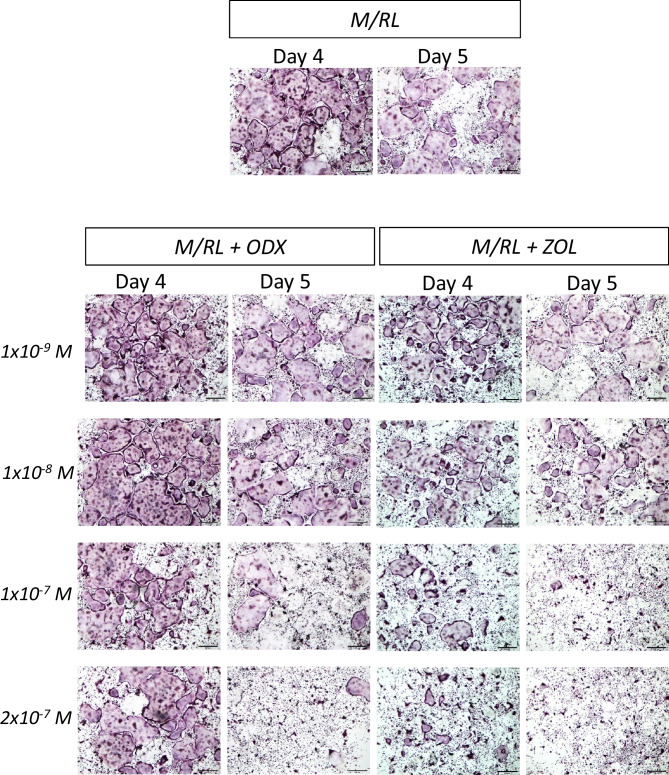
Osteodex (ODX) and zoledronic acid (ZOL) enhance mature osteoclast cell death in a concentration-dependent manner. Bone marrow macrophages were incubated in the presence of M-CSF (M; 30 ng/ml) and RANKL (RL; 4 ng/ml) with or without different concentrations of ODX or ZOL. Cells were stained for TRAP after 4 and 5 days

We next assessed the effect by different concentrations of ODX on mature osteoclasts. On day 4, no effect by ODX at 1 × 10^−9^ M and 1 × 10^−8^ M was observed. At 1 × 10^−7^ M and 2 × 10^−7^ M, however, a modest increase of dead mature osteoclasts could be observed (Fig. 5). At day 5, ODX at 1 × 10^−7^ and 2 × 10^−7^ M clearly had enhanced the number of dead osteoclasts compared to M-CSF/RANKL without bisphosphonates, a difference which was more modest at 1 × 10^−8^ and absent at 1 × 10^−9^ M (Fig. 5).

The effect by ZOL on mature osteoclasts in the BMM was concentration-dependent (Fig. 5). At day 4, ZOL at 1 × 10^−9^ M had a modest effect, and then the response gradually was more evident at 1 × 10^−8^ M, 1 × 10^−7^, and 2 × 10^−7^ M. At day 5, effects were more pronounced than at day 4 at all concentrations tested.

**Fig. 6:**
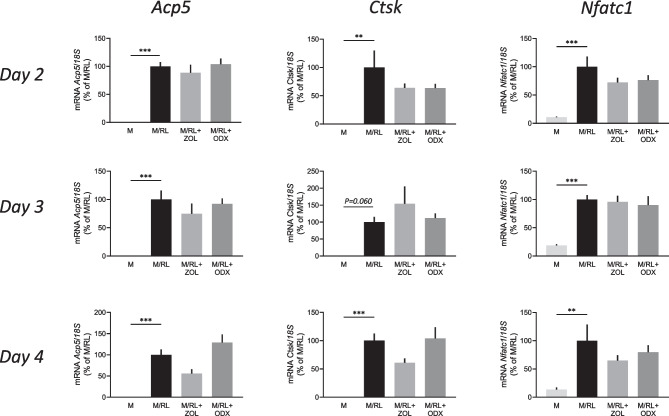
Osteodex (ODX) and zoledronic acid (ZOL) do not affect the expression of osteoclastic (*Acp5, Ctsk*) and osteoclastogenic (*Nfatc1*) genes induced by RANKL. Bone marrow macrophages were incubated in the presence of M-CSF (M; 30 ng/ml) and RANKL (RL; 4 ng/ml) with or without ODX or ZOL, both at 2 × 10^−7^ M. After 2, 3, and 4 days, RNA was extracted, and gene expression was analyzed. Data are means of four observations, and SEM is given as vertical bars. Asterisks denote statistical significance; **P < 0.01 and ***P < 0.001, one-way ANOVA, followed by Dunnett’s multiple comparisons test versus M/RL

These observations indicate that ODX, similar to ZOL, decreases osteoclast numbers by inducing cell death in mature osteoclasts without affecting the differentiation of mononucleated osteoclast progenitor cells. However, the cell death response seems slightly delayed and slightly less potent compared to ZOL. To further confirm that ODX does not affect the differentiation of osteoclast progenitor cells, we next analyzed the expression of genes in BMM known to be associated with osteoclastogenesis. M-CSF/RANKL stimulation induced the mRNA expression of *Acp5* (encoding TRAP) and *Ctsk* (encoding cathepsin K), as expected (Fig. 6). The expression of these osteoclastic genes was not significantly affected by ODX or ZOL at early (day 2) or late stages (days 3 and 4) of osteoclastogenesis. This finding suggests that ODX and ZOL did not interfere with osteoclastogenic signaling mechanisms downstream the receptor RANK. This conclusion was further confirmed by the observation demonstrating that M-CSF/RANKL-induced upregulation of the mRNA expression of the osteoclastogenic transcription factor *Nfatc1* was not affected by ODX or ZOL (Fig. 6).

**Fig. 7:**
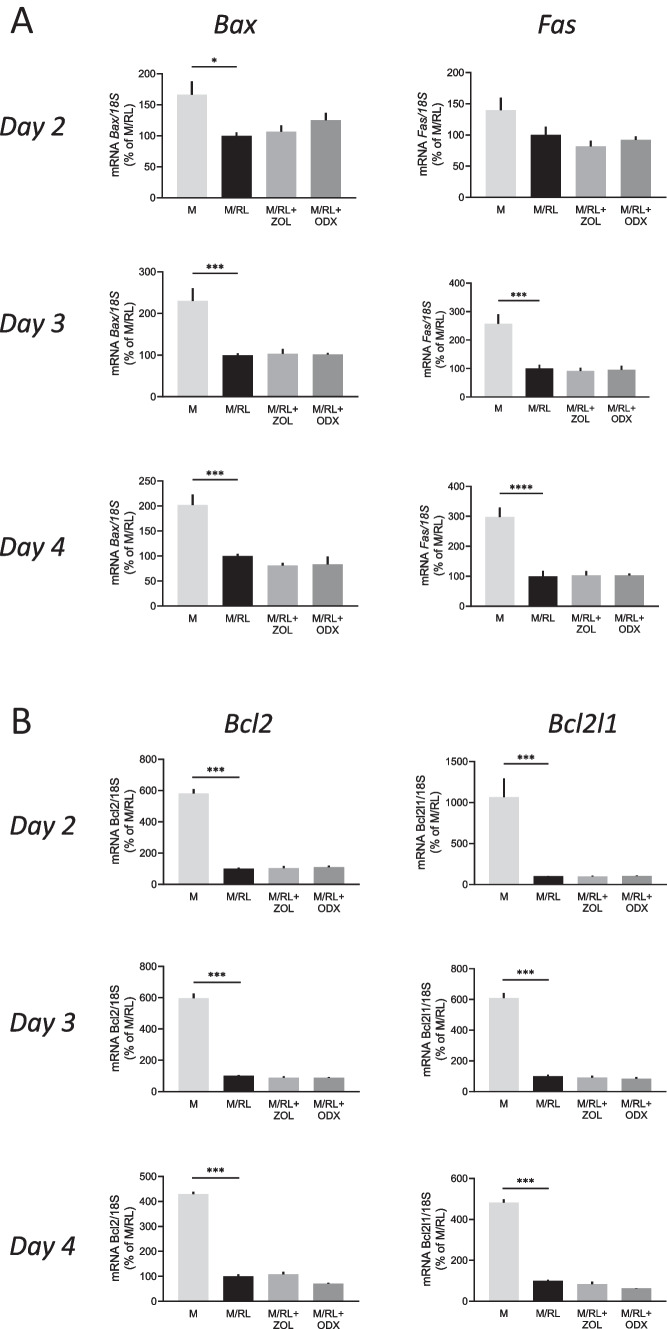
Osteodex (ODX) and zoledronic acid (ZOL) do not affect the expression of pro-apoptotic (**A**) and anti-apoptotic (**B**) genes regulated by RANKL. Bone marrow macrophages were incubated in the presence of M-CSF (M; 30 ng/ml) and RANKL (RL; 4 ng/ml) with or without ODX or ZOL, both at 2 × 10^−7^ M. At the stated time periods, RNA was extracted, and gene expression was analyzed. Data are means of four observations, and SEM is given as vertical bars. Asterisks denote statistical significance; **P < 0.01 and ***P < 0.001, one-way ANOVA, followed by Dunnett’s multiple comparisons test versus M/RL

To assess if the activities by ODX and ZOL on mature osteoclast cell death were associated with regulation of anti- or pro-apoptotic genes, we analyzed the mRNA expression of four such genes. The *Bax* and *Fas* genes, known to be pro-apoptotic, were both significantly downregulated by RANKL (Fig. 7A, B). This response was not affected by ODX or ZOL (Fig. 7A, B). The *Bcl2* and *Bcl2l1* genes are known to be anti-apoptotic, and both were significantly downregulated by RANKL, responses also unaffected by ODX or ZOL (Fig. 7C, D).

## Discussion

ODX is a polymer based on a carbohydrate backbone with alendronate and guanidine moieties having anti-tumor and anti-resorptive capacity [18]. ODX has been investigated in clinical trials (phase I, phase II) for the treatment of bone metastases in castration-resistant prostate cancer. The results confirm a profound inhibitory effect on bone markers, primary on osteoclast markers and secondary on osteoblast markers. Direct anti-tumoral effects were recorded without significant side effects [30, 31]. We here demonstrate that ODX exerts anti-resorptive activity by enhancing mature osteoclast cell death without affecting the differentiation of osteoclast progenitor cells to mature osteoclasts.

We have previously reported that ODX inhibits bone resorption in ex vivo cultures of neonatal mouse calvaria, resulting in decreased calcium release from the explants. Here, we demonstrate that this response is associated with a robust decrease of multinucleated osteoclast numbers. The stimulator used, ATRA (the biologically active metabolite of vitamin A [26]), increases osteoclast formation and bone resorption indirectly through enhanced production of the osteoclastogenic cytokine RANKL [27]. RANKL can be produced by several cell types including osteoblasts and osteocytes and binds to the cognate receptor RANK on mononuclear osteoclast progenitor cells to induce their differentiation to mature, multinucleated osteoclasts [32]. The decoy receptor osteoprotegerin (OPG) also binds to RANKL and inhibits the binding to RANK. We found that ODX did not affect the mRNA expression of *Tnfsf11* (encoding RANKL) or *Tnfrsf11b* mRNA expression (encoding OPG) indicating that ODX inhibited osteoclast formation through a direct action on either osteoclast progenitors or mature osteoclast.

Periosteal cells isolated from neonatal mouse calvariae, often designated mouse calvarial osteoblasts, are enriched with osteoblasts but also contain a substantial amount of macrophages/osteoclast progenitor cells which will form osteoclasts when stimulated with RANKL or by osteoclastogenic cytokines and hormones enhancing the expression of RANKL in osteoblasts [21]. Recent single-cell RNA sequencing has also demonstrated the presence of macrophages in these isolations and that their numbers enhance during cell culture [22]. To gain further support to the conclusion that ODX targets cells in the osteoclastic lineage, we stimulated calvarial periosteal cell cultures with RANKL with and without ODX. The finding that ODX abolished RANKL-stimulated osteoclast formation further shows that ODX can inhibit osteoclast formation independent of RANKL/OPG production, although these experiments cannot demonstrate if ODX exerts its anti-osteoclastogenic effect by inhibiting osteoclast differentiation or promoting mature osteoclast cell death.

The experiments using calvarial explants and calvarial cells indicate that ODX can decrease the number of osteoclasts on the cortical periosteum. We used bone marrow cell cultures to assess whether ODX can inhibit osteoclast formation also on endosteal surfaces or on trabecular bone. We found that ODX robustly inhibited osteoclast formation also in these cell cultures.

The common standard view is that inhibition by BPs of bone resorption in vivo is due to the binding of BPs to bone mineral through its affinity to hydroxyapatite crystals and that osteoclasts are exposed to high concentrations of BPs when the mineral crystals are dissolved during the resorptive process [28]. This is the reason why one injection of ZOL per year is sufficient for the treatment of patients with osteoporosis. The reduction of mature osteoclasts observed in cell cultures on plastic dishes due to ODX illustrates the ability of ODX to impede mature osteoclasts irrespective of its binding to mineral crystals and without requiring active bone resorption by osteoclasts.

To investigate if ODX inhibited osteoclast formation in the cell cultures by interfering with osteoclast differentiation or by enhancing mature osteoclast cell death, we next purified macrophages from bone marrow (BMM) and used them as osteoclast progenitor cells [23, 33]. Since all cells in these cultures express the macrophage marker CD11b as assessed by FACS analysis [33], ODX can only target cells in the macrophage/osteoclast lineage in these cultures. Similar to the findings in bone marrow cell cultures, ODX robustly decreased the number of osteoclasts in the BMM cultures as demonstrated in TRAP-stained cultures and by staining of the characteristic actin ring in mature osteoclasts. When BMM cells were incubated on bone slices, ODX robustly inhibited the release of CTX from bone slices, demonstrating the anti-resorptive effect by ODX although this analysis cannot discriminate between inhibition of osteoclast formation and stimulation of osteoclast cell death. Osteoclast counting was not performed in these experiments since the continuous fusion of mono- and multinucleated osteoclasts to huge, pancake-like osteoclasts, which gradually become apoptotic, makes counting of the numbers of osteoclasts not an accurate measure.

The observation that ODX did not affect RANKL-induced upregulation of mRNA expression of the osteoclastic genes *Acp5* and *Ctsk* during the first 4 days in the BMM cultures, a time period when the mononuclear osteoclast progenitors differentiate to mature osteoclasts, demonstrates that ODX does not affect osteoclast progenitor cell differentiation. Intracellular signaling downstream activated RANK includes activation of MAPK and transcription factors such as AP-1, NF-κB, PU.1, MITF, and NFATc1, with NFAC1 being considered the master regulator of osteoclastogenesis [34]. The fact that ODX did not affect RANKL-induced upregulation of *Nfatc1* mRNA expression shows that ODX does not affect RANK signaling upstream Nfatc1. Similarly, ZOL did not affect the mRNA expression of osteoclastic and osteoclastogenic genes.

In all three assay systems used, the effects by ODX were similar to those obtained by ZOL, a well-documented and clinically often used BP. Since ODX, similar to ZOL, is a nitrogen-containing BP and since this class of BP exerts its anti-osteoclastic effects through stimulating mature osteoclast apoptosis [28], we made detailed observations on osteoclast morphology at different time points during the culture. It was evident that ODX had no effect on mature osteoclast numbers at early time points (days 3 and 4) but clearly at day 5, also demonstrating that ODX does not affect osteoclast differentiation and formation of mature osteoclasts. The remnants of osteoclasts observed at late time points had the appearance of apoptotic osteoclasts with parts of the cell membrane, nuclei, and cytosolic compartments persisting, although formal proof for that ODX caused mature osteoclast death by apoptosis would require more detailed analyses. This response was very sensitive and observed at concentrations of ODX at 0.01 µM and above, which is considerably lower than those usually used to study effects by BPs on apoptosis in vitro where concentrations in the range of 10–100 µM often are used [17, 35].

We assessed the mRNA expression of four pro- and anti-apoptotic genes regulated by RANKL, but none was affected by ODX, which suggests that ODX-induced cell death is induced by other mechanisms. This observation further supports our conclusion that ODX does not affect RANK signaling. Both nitrogen-containing BPs and non-nitrogen BPs inhibit osteoclasts, but only nitrogen-containing BPs inhibit the mevalonate pathway [17]. Nitrogen-containing BPs inhibit the incorporation of ^14^C-mevalonate into both farnesylated and geranylgeranylated GTP-binding proteins in rabbit osteoclasts [17]. The fact that GGTI-298, a specific inhibitor of geranylgeranyl transferase I, induces osteoclast apoptosis indicates that geranylgeranylation of proteins is more important than farnesylation of proteins for osteoclast function.

In conclusion, we here report that ODX does not inhibit mature osteoclast formation but inhibits osteoclastic bone resorption by decreasing osteoclast numbers through enhanced cell death of mature osteoclasts. ODX and ZOL seem to be equipotent as inhibitors of bone resorption [18] and inducers of osteoclast cell death (present study). Important from a clinical point of view is the observation that ODX is considerably more potent than ZOL as inducer of apoptotic cell death in human prostate cancer and breast cancer cell lines [18]. These findings indicate that ODX causes apoptosis in tumor cells and cell death of mature osteoclasts by different mechanisms.

## Data Availability

The datasets generated and analyzed during the current study are available from the corresponding author on reasonable request.

## Abbreviations

ATRA: All-*trans*-retinoic acid
BMC: Bone marrow cells
BMM: Bone marrow macrophages
BP: Bisphosphonates
FCS: Fetal calf serum
FFPS: Farnesyl diphosphate synthase
M-CSF: Macrophage colony-stimulating factor
α-MEM: α-Modification of minimum essential medium
ODX: Osteodex
OPG: Osteoprotegerin
RANK: Receptor activator of NF-κB
RANKL: Receptor activator of NF-κB ligand
SRE: Skeletal related events
TRAP: Tartrate-resistant acid phosphatase
TRAP^+^MuOCL: TRAP^+^ multinucleated osteoclasts
D3: 1,25(OH)_2_-vitamin D3
ZOL: Zoledronic acid
